# HYPERTENSION DOES NOT CONTRIBUTE TO MICROBLEEDS ONSET IN FEMALE EFAD MICE

**DOI:** 10.1093/geroni/igz038.352

**Published:** 2019-11-08

**Authors:** Mafalda Cacciottolo, Todd E Morgan, Caleb E Finch

**Affiliations:** 1 University of Southern California, Los Angeles, California, United States; 2 Leonard Davis School of Gerontology, University of Southern California, Los Angeles, California, United States

## Abstract

Cerebral microbleeds (MBs) contribute to pre-clinical cognitive decline and are an additional clinical burden in Alzheimer Disease (AD). Hypertension is associated with MBs, with nearly 2-fold higher likelihood for MBs per SD increment in blood pressure (BP). We investigated the possible role of age-related hypertension in the EFAD mouse model (transgenic for carrying familial AD mutations and targeted replacement of human APO-E3 or -E4). MBs were detected by Prussian Blue histochemistry. We extended prior findings with observations that MBs arise early in life, by 2 months, and confirmed female excess for ApoE3 and-E4 carriers. Wildtype C57BL/6J mice also accumulated MBs, and a 10-fold lower level and more slowly up to 21mo of age. BP was measured by the tail-cuff method. All mice had BP in the normotensive range, <150 mm Hg, systolic. Longitudinal measurements of blood pressure at ages 2, 4, and 6 months showed few age changes, except for E3FAD females at 6 months (systolic, +20%, p<0.05; diastolic, +33%, p<0.05). A possible decrease in blood pressure was observed in EFAD mice (-33%, p<0.01) compared to C57BL/6J mice. A not statistical trend of increase was observed in older C57BL/6J mice up to 18 mo of age, consistent with previous reports. Older ages are required for complete negation of role of hypertension in the MB model. Ongoing studies will examine mice older than 6 months for potential relations of blood pressure and MB, and in relation to brain amyloid deposits which surrounded MBs in our prior study.

